# New Insights into Cd^2+^/Fe^3+^ Co-Doped BiOBr for Enhancing the Photocatalysis Efficiency of Dye Decomposition under Visible-Light

**DOI:** 10.3390/nano11020423

**Published:** 2021-02-07

**Authors:** Hong Sheng, Wei Wang, Rong Dai, Jing Ning, Lei Zhang, Qiao Wu, Fuchun Zhang, Junfeng Yan, Weibin Zhang

**Affiliations:** 1 College of Mathematics & Physics, Weinan Normal University, Weinan 714000, China; wnshenghong@163.com; 2 School of Physics and Electronic Information, Yan’an University, Yan’an 716000, China; wangwei@yau.edu.cn (W.W.); dairong@yau.edu.cn (R.D.); ningjing@yau.edu.cn (J.N.); yadxzl960203@163.com (L.Z.); wq@yau.edu.cn (Q.W.); 3 School of Information Science Technology, Northwest University, Xi’an 710127, China; 4 School of Physics and Optoelectronic Engineering, Yangtze University, Jingzhou 434023, China

**Keywords:** BiOBr, Cd^2+^/Fe^3+^-doping, density function theory, microsphere, photocatalytic activity

## Abstract

Uniform flowerlike microspheres Cd^2+^/Fe^3+^ co-doped BiOBr were prepared with the aid of the microwave hydrothermal process. The results indicate that the degradation performance of Bi_1−*x*_Cd*_x_*OBr and Bi_1−*x*_Fe*_x_*OBr are 1.31 and 2.05 times that of BiOBr for RhB, respectively. Moreover, the novel Cd^2+^/Fe^3+^ co-doped BiOBr photocatalysts with ~0.42 eV impurity bands presented remarkably enhanced photocatalytic activities with being 3.10 times that of pure BiOBr, by achieving e^−^/h^+^ efficient separation and narrowed bandgap with the ions synergistic effect of Cd^2+^ and Fe^3+^. Based on DFT insights, the photodegradation mechanism was systematically studied that the conversion of multivalent Fe^3+^ ions promoted the production of •O_2_^−^, and Cd^2+^ ions worked as electron transfer mediators, which elucidated that the •O_2_^−^ and h^+^_VB_ mainly participated in the catalytic reaction. The experimental and theoretical results show that the synergistic effects of multi-ion doping have great potential in the field of photocatalysis.

## 1. Introduction

In the past decades, with the increase of volumes of polluted water, the effective treatment of polluted water has become an urgent problem [1,2,3]. Organic pollutants are one of the main pollutants; although there are many methods to deal with them, there are still many limitations to solve the problem, which has always been the driving force for the development of water purification technology with low-cost and high-efficiency, so as to solve serious environmental problems and meet the government’s environmental requirements. As early as 1972, Honda and Fujishima proposed that TiO_2_ with single-crystal electrodes as photocatalysts could be applied to decompose water under ultraviolet irradiation [4,5,6]. Since then, photocatalysis, with economic, efficient, and environmental characteristics, has attracted researchers’ attention in the wastewater treatment field.

Some of the existing oxide semiconductor materials (i.e., TiO_2_, ZnO) have been utilized in the field of photocatalysis, and some researchers have paid more effort to modify TiO_2_ [5,7,8] and study non-TiO_2_ semiconductors [9,10,11]. Unfortunately, these oxide semiconductors, with a high bandgap of above 3.0 eV, can only be activated under ultraviolet light, which occupies less than 5% of the solar spectrum [12,13]. To be precise, photocatalysts with a larger bandgap exhibit lower utilization of the sunlight spectrum under the same conditions. With more understanding of semiconductor materials and photocatalysis mechanism, the application of semiconductor materials in the photocatalysis field has made dramatic progress, which also contributes to environmental protection and energy conservation.

Currently, the semiconductor photocatalysts with exposed different crystal planes have been studied, and it has been proved that different exposed crystal planes would result in different electron structures, which would further lead to various energy band levels. More importantly, the energy band levels of the semiconductor materials directly affect their photocatalytic performance [14,15,16]. Hence, layered Bi-based photocatalysts have become a hot topic. The BiOX (X = Cl, Br, I), exhibiting potential value and prospects in The field of photocatalysis, has been widely investigated [17,18,19,20,21]. BiOX has been proved to be an ideal carrier for heterogeneous catalytic reactions because of its visible light response, high chemical stability, etc. [22,23]. The doping process with rare-earth atoms and transition metals (TMs) in materials is considered a new way to promote the photocatalytic performance of materials. The doping can create vacancies or defects and change the bandgap in BiOX materials. It can lead to modifications to the intrinsic properties of materials by redistribution of electrons. The doped impurity atoms can provide impurity energy levels and change charge transfer properties of materials, leading to enhanced performance in some catalytic reactions [24,25,26,27,28].

A soft chemical method was applied to fabricate BiOBr oxyhalide photocatalysts, which exhibited excellent photocatalytic ability in the light [29]. Hu et al. [30] successfully prepared Bi_1−x_Ce_x_OBr via the hydrothermal process. It can be seen that the morphology of samples gradually changed with the increase of Ce^3+^ doping amount, and a blueshift occurred by increasing the bandgap of samples. Liu et al. [31] synthesized Bi_1−*x*_Al*_x_*OBr with different Al^3+^ content with a solvothermal process. The high photocatalytic performance was considered to arise due to the separation of e^−^-h^+^ and enough active sites. Ti-doped BiOBr photocatalyst was prepared by a two-component process by Wang et al. [32]; the photocatalytic performance of Ti-doped BiOBr was improved by increasing BET surface area. The La^3+^-doped BiOBr was prepared [33], and the high photocatalytic performance of samples was attributed to the effective separation of e^−^-h^+^ pairs and the narrow bandgap. The holes were considered as the main active substances. The homogeneous porous Fe^3+^ and Er^3+^ ions co-doped Bi_5_O_7_I (BiOI) microspheres were synthesized via the solvothermal decomposition by Liu et al. [34]. Yuan et al. [22] successfully prepared Fe (III)-modified BiOBr via a facile one-step route. Moreover, they believed that the H_2_O_2_ enhanced the performance of organic dye degradation and benzyl alcohol oxidation. Liu et al. [35] synthesized Fe^3+^ doped BiOBr based on Jace micromotor and explained the excellent photocatalytic performance under mild pH conditions and concentration of H_2_O_2_. Huang et al. [36] synthesized hierarchical Fe^3+^-modified BiOCl micro-flowers by a one-step solvothermal method, which demonstrates that Fe^3+^-modified BiOCl plays an important role in promoting the degradation of the gaseous decomposition acetaldehyde. Though Cd^2+^ is highly toxic [37], Cd^2+^ ions not only change the properties of the original semiconductors but also has a great influence on the whole catalytic activity with the increase of doping amount [38,39]. In addition to controlling the growth of crystal form and the size of microcrystalline, the surface structure, spectral response range and bandgap energy of the original semiconductor are further changed by introducing Cd^2+^ ions via chemical method [40]. No previous studies about the preparation of Cd^2+^/Fe^3+^ co-doped BiOBr flowerlike microspheres for the photo-decomposition of dye were found.

In this work, Cd^2+^/Fe^3+^ co-doped BiOBr flowerlike microspheres were first prepared by a hydrothermal process and investigated the photocatalytic performance of products by the degradation of dye. Based on density functional theory (DFT), the enhanced photocatalytic activities of BiOBr by Cd^2+^/Fe^3+^-doping were discussed in detail. Furthermore, the possible growth mechanism and photocatalysis mechanism of Cd^2+^/Fe^3+^ co-doped BiOBr were investigated in the work.

## 2. Experimental

### 2.1. Materials

The chemical reagents are all analytical grade in this work. Bismuth nitrate pentahydrate (Bi(NO_3_)_3_·5H_2_O) was obtained from Klamar (Shanghai, China). Hexadecyl trimethyl ammonium bromide (CTAB), cadmium nitrate (Cd(NO_3_)_2_·4H_2_O), iron(III) nitrate nonahydrate (Fe(NO_3_)_3_·9H_2_O) and ethylene glycol((CH_2_OH)_2_) were purchased from Kermel (Tianjin, China).

### 2.2. Catalyst Preparation

First of all, Bi(NO_3_)_3_·5H_2_O and CTAB with 1:1 molar ration (4 mmol) were dissolved in 20 mL ethylene glycol (EG) with ultrasonication for 15 min, respectively. After 15 min of magnetic stirring respectively, the above solutions were mixed for 30 min. Then it was transferred to the Teflon-lined autoclave. Then it was reacted at 180 °C for 15 min. After this, the gray products were cleaned by deionized water (DI) several times, last washed by alcohol for once, and dried. The Cd^2+^-doped BiOBr and Fe^3+^-doped BiOBr microspheres were synthesized by the above identical experimental procedure with the addition of 2 wt % Cd(NO_3_)_2_·4H_2_O or 2 wt % Fe(NO_3_)_3_·9H_2_O in the reaction solution, which was denoted as Bi_1−*x*_Cd*_x_*OBr and Bi_1−*x*_Fe*_x_*OBr (*x* = 0.02). The synthesis of Cd^2+^/Fe^3+^ co-doped BiOBr microspheres, denoted as Bi_1−*x*−*y*_Cd*_x_*Fe*_y_*OBr (*x* = *y* = 0.02), also performed with the same procedure as Cd(NO_3_)_2_·4H_2_O, with the addition of 2 wt % Fe(NO_3_)_3_·9H_2_O into the reaction solution.

### 2.3. Growth Mechanism

**Scheme 1:**
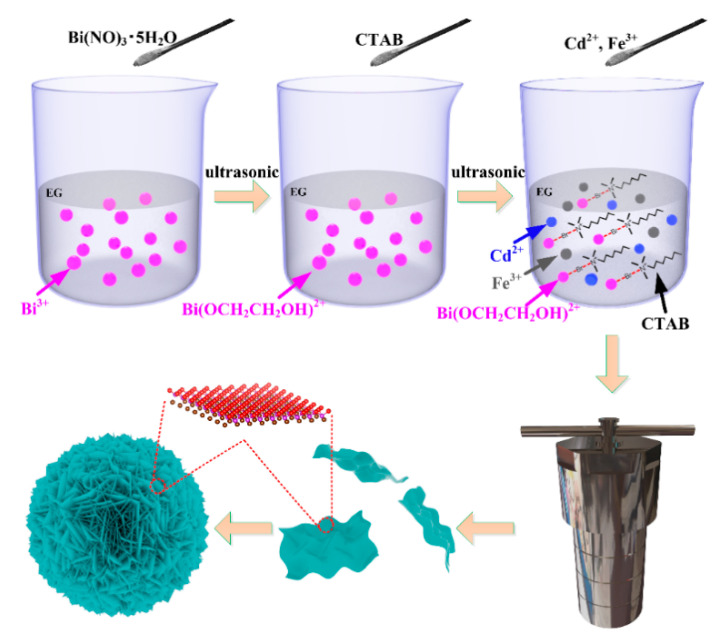
The preparation steps of flowerlike Cd^2+^/Fe^3+^ co-doped BiOBr with hexadecyl trimethyl ammonium bromide (CTAB) as bromine source.

The growth mechanism of flowerlike microsphere BiOBr with CTAB as Br source and the effect of 3d transition metal doping were understood clearly as the purpose. The possible growth mechanism of flowerlike microsphere Cd^2+^/Fe^3+^ co-doped BiOBr photocatalyst, composing of ultrathin nanosheets, could be proposed according to Scheme 1. After being ultrasonicated, the Bi(NO_3_)_3_·5H_2_O was completely dissolved in EG with ultrasonication, and many Bi^3+^ ions were produced, as shown in Formula (1). Meanwhile, the alkoxides (Bi(OCH_2_CH_2_OH)^2+^) formed by coordination of EG with Bi^3+^ (as shown in Formula (2)), which was linearly aligned structure and stable as a dense [41,42]. After this, the CTAB was added into the above solution containing Bi^3+^ as the Bi source and ultrasonicated to dissolve completely. The CTAB surfactant, acting as both Br source and template, was forming the lamellar structure [43]. Subsequently, the combination of Br^−^ in CTAB lamellas with Bi(OCH_2_CH_2_OH)^2+^ induced the formation of a stable chain structure on the template provided by CTAB (as shown in Scheme 1). In addition, the 2 wt % Cd^2+^ and 2 wt % Fe^3+^ were then added to the above solution. As shown in Formula (3), the flowerlike microsphere Cd^2+^/Fe^3+^ co-doped BiOBr consists of ultrathin nanosheets. The Cd^2+^/Fe^3+^ co-doped BiOBr nucleated grew anisotropically and shaped into the lamellar shape with CTAB as the template (as shown in Scheme 1). Additionally, the layered molecular structure of the sample (ball stick model) was also given in Scheme 1. In the beginning, the crystalline nucleus was formed in a supersaturated medium, and then the crystal growth followed. In hydrothermal reaction, enough energy (180 °C in our reaction system) input overcomes the reaction barrier, so the inherent anisotropic growth habit can happen. The ion radius of both Fe^3+^ (0.645 Å) and Cd^2+^ (0.97 Å) ions are smaller than that of Bi^3+^ (1.03 Å) ion, and that of Fe^3+^ ion is the smallest. As a result, Fe and Cd atoms can easily replace Bi atoms and form a new stable structure:

(1) $$\mathrm{Bi}{\left({\mathrm{NO}}_{3}\right)}_{3}\cdot 5H_{2}O\;\to {\;\mathrm{Bi}}^{3+}+3{\mathrm{NO}}_{3}^{-}$$

(2) $${\mathrm{Bi}}^{3+}+{\mathrm{HOCH}}_{2}{\mathrm{CH}}_{2}\mathrm{OH}\to \;\mathrm{Bi}{\left({\mathrm{OCH}}_{2}{\mathrm{CH}}_{2}\mathrm{OH}\right)}^{2+}+H^{+}$$

(3) $$\mathrm{Bi}{\left({\mathrm{OCH}}_{2}{\mathrm{CH}}_{2}\mathrm{OH}\right)}^{2+}+{\;C}_{16}H_{33}{\left({\mathrm{CH}}_{3}\right)}_{3}N-\mathrm{Br}\;+\;+{\mathrm{xCd}}^{2+}+{\mathrm{yFe}}^{3+}\;\;\;\;\;\to {\mathrm{Bi}}_{1-x-y}{\mathrm{Cd}}_{x}{\mathrm{Fe}}_{y}\mathrm{OBr}↓+{\;C}_{16}H_{33}{\left({\mathrm{CH}}_{3}\right)}_{3}N^{+}+H_{2}O+{\;H}^{+}$$

### 2.4. Characterization

X-ray diffraction (XRD), operating at 40 kV and 30 mA, and the scanning rate of 2 degrees/min, was employed to identify the phase of specimens. Transmission electron microscope (TEM, JEOL, Tokyo, Japan) was employed to investigate the micromorphology. The morphology of as-prepared specimens was detected by a scanning electron microscope (SEM, SIGMA, St. Louis, MO, USA) operating at 3 kV. The energy-dispersive X-ray spectroscopy (EDS, JEOL, Tokyo, Japan) was employed to describe the elemental mappings of photocatalysts. The X-ray photoelectron spectroscopy (XPS, Kratos, Manchester, UK) were applied to obtain types and valence states of elements. The absorption spectra were measured by an ultraviolet-visible-near-infrared spectrometer (UV-vis, Metash, Shanghai, China) with fast scanning speed; the conversion wavelength of the light source was 310 nm, the slit width was 20 nm, the conversion wavelength of the grating was 720 nm, and the data interval is 1.0 nm. The molecular vibration information of specimens was characterized by a Raman spectrometer. The photoluminescence (PL) spectra of products were obtained by spectrophotometer exciting at 325 nm.

### 2.5. Photocatalytic Tests

The photocatalytic performance of X-doped BiOBr (X = Cd, Fe, Cd/Fe) was estimated by obtaining the degradation of Rhodamine B (RhB) under visible light irradiation at ambient temperature. A 500 W long arc xenon lamp with AM 1.5 filter (100 mW·cm^−2^) was employed to simulate the visible light. The 50 mg of the catalyst was dispersed uniformly into 50 mL RhB solution (RhB = 10 mg·L^−1^) in a quartz catalyst tube to carry out the decompose of RhB under visible light irradiation. The above RhB solution with different samples was continuously stirred for 30 min to ensure an adsorption–desorption equilibrium in the dark. The 5 mL of suspension was taken out every 15 min during the irradiation and analyzed by using a UV1901PC ultraviolet-visible spectrophotometer. The wavelength of RhB was detected at 556 nm. The degradation rate (*η*) of RhB was evaluated by the following formula:

*η* = (*C*_0_/*C*)/*C*_0_ (4)

where *C* means the RhB concentrations at different intervals and *C*_0_ is the initial concentration, respectively.

## 3. Results and Discussion

**Figure 1:**
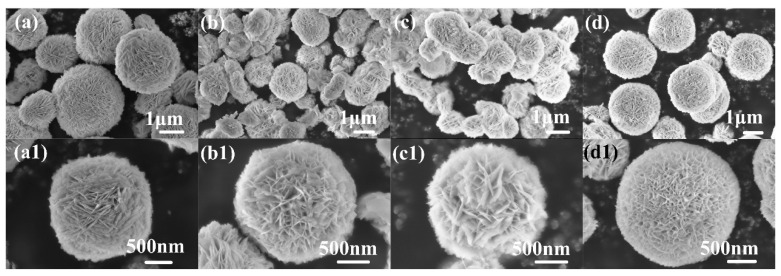
FESEM and HRSEM images of BiOBr (**a**,**a1**), Bi_1−*x*_Cd*_x_*OBr (**b**,**b1**), Bi_1−*x*_Fe*_x_*OBr (**c**,**c1**), Bi_1−*x*−*y*_Cd*_x_*Fe*_y_*OBr (**d**,**d1**).

Figure 1 shows the SEM images of pure-BiOBr and X-doped BiOBr (X = Cd, Fe, Cd/Fe). As shown in Figure 1a, the pure-BiOBr presents a uniform morphology, and the average diameter is 2–3 μm. Figure 1(a1) shows HRSEM images of BiOBr. The porous nanospheres formed through stacking a large number of nanosheets can be observed. The SEM diagrams of Bi_1−*x*_Cd*_x_*OBr (Figure 1(b,b1)), Bi_1−*x*_Fe*_x_*OBr (Figure 1(c,c1)) and Bi_1−*x*−*y*_Cd*_x_*Fe*_y_*OBr (Figure 1(d,d1)) are shown, respectively. The average diameter of Bi_1−*x*_Cd*_x_*OBr and Bi_1−*x*_Fe*_x_*OBr microspheres is about 1.5–2 μm. In addition, the incorporation of Cd^2+^ and Fe^3+^ ions have made BiOBr microspheres lose, comparing with that of pure-BiOBr. However, it can be seen that the particle size of Cd^2+^/Fe^3+^ co-doped BiOBr is almost the same as that of pure-BiOBr, and the morphology is uniform and dense. The possible reason for this may be the joint effect of Cd^2+^ and Fe^3+^ ions in the BiOBr lattice. It can be seen (Figure 1(b1,c1,d1)) that the porous structure of X-doped BiOBr (X = Cd, Fe, Cd/Fe) is particularly obvious, and the dispersion and crystallinity of X-doped nanosheets are better than that of pure-BiOBr (Figure 1(a1)), which agrees with the XRD. In addition, the surfaces of X-doped BiOBr (X = Cd, Fe, Cd/Fe) are composed of thin nanosheets, which form porous structures with a large specific surface area. The layered structure of X-doped BiOBr (X = Cd, Fe, Cd/Fe) provides possibilities for atoms to promote the separation of e^−^/h^+^ pairs. Among all samples, the looseness of nanosheets composed of Bi_1−*x*−*y*_Cd*_x_*Fe*_y_*OBr is the highest. Consequently, the photocatalytic properties of Cd^2+^/Fe^3+^ co-doped BiOBr are better than that of single doping [44].

**Figure 2:**
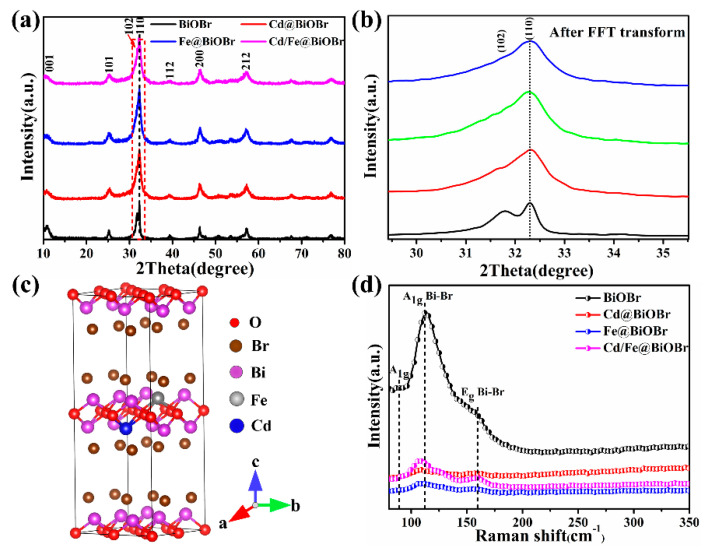
XRD patterns of samples (**a**), enlarged XRD patterns with FFT transform (**b**), the crystalline structure model of the X-doped BiOBr (X = Cd, Fe, Cd/Fe) (**c**), and Raman spectra (**d**).

XRD patterns of as-prepared samples are presented in Figure 2a, all peaks of samples can be assigned to BiOBr (JCPDS 09-0393) [45]. Moreover, no other peaks appeared. The results indicate that the doping of Cd^2+^ and Fe^3+^ ions have no obvious effect on the structure of the matrix. The peaks of X-doped BiOBr (X = Cd, Fe, and Cd/Fe) are all stronger than that of BiOBr (Figure 2a), which illustrates that the doping of Cd^2+^ and Fe^3+^ can improve the crystallinity of samples. The (102) and (110) peaks are enlarged after Fourier transform processing, shown in Figure 2b: most of the signals, especially the (110) peak became more obvious, (102) peak weakened gradually, which is attributed to Fe–O, Cd–O, and Cd–Br. Consequently, the growth crystal plane along with the (110) crystal plane is enhanced simultaneously, and the (102) plane is inhibited. The crystalline structure model of the X-doped BiOBr (X = Cd, Fe, Cd/Fe) is shown in Figure 2c, indicating no obvious change in the structure. The Raman spectra of as-prepared products are shown in Figure 2d to acquire the detailed information of Cd^2+^ and Fe^3+^-doped BiOBr on the chemical bonds. The main peak of pure-BiOBr centered with 112.3 cm^−2^ can be seen in Figure 2d, which is attributed to the A_1g_ stretching mode of Bi-Br bond. The internal Eg stretching mode of the Bi-Br bond can be described as the peak of 160.3 cm^−2^ [46]. Notably, there is a peak at 86.6 cm^−2^, which may be attributed to the first-order vibration of A_1g_ during the growth of BiOBr [47]. The peak intensity of Cd^2+^ and Fe^3+^-doped BiOBr is lower than that of BiOBr, indicating that new chemical bonds formed. The stretching of the A_1g_ and Eg chemical bonds of the Bi-Br bond were inhibited, and the charge transfer characteristics changed in the BiOBr crystal lattice [31]. It is found that the Raman spectra of Cd^2+^ and Fe^3+^-doped BiOBr shift to the left, and the Raman spectra of Cd^2+^/Fe^3+^ co-doped BiOBr shift ~5.5 cm^−^^1^, which attribute to the bond energy of Cd and Fe atoms with small atomic radius is greater than that of Bi-O when they form a new bond with O. In addition, different chemical bonds emerge different normal vibration with Raman activity when the laser is shining on the sample surface, and the polarization also change.

**Figure 3:**
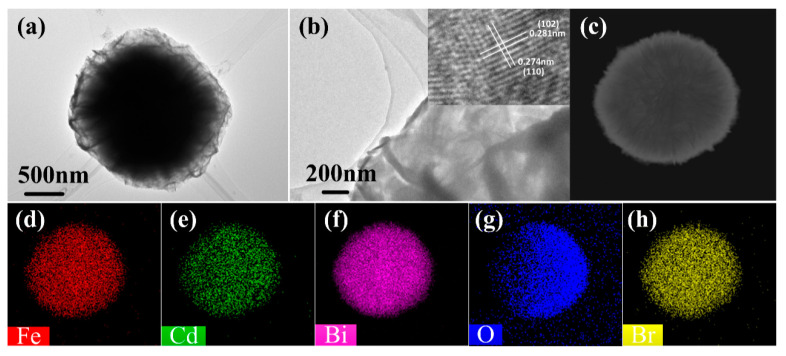
TEM image of Bi_1−*x*−*y*_Cd*_x_*Fe*_y_*OBr (**a**); HRTEM image of Bi_1−*x*−*y*_Cd*_x_*Fe*_y_*OBr (**b**) and corresponding elemental mappings of Bi_1−*x*−*y*_Cd*_x_*Fe*_y_*OBr (**c**–**h**).

The microstructure of the Bi_1−*x*−*y*_Cd*_x_*Fe*_y_*OBr microspheres was characterized by TEM (Figure 3a). The samples, assembled by nanosheets, present the quasi-microsphere-like structure. The average size of the microspheres is about 2–3 μm. It can be seen that the edge of samples shows some nanosheets (shown in Figure 3b). High-resolution TEM (HRTEM) images (illustration in Figure 3) of Bi_1−*x*−*y*_Cd*_x_*Fe*_y_*OBr shows that the lattice fringe spacing of 0.281 and 0.274 nm ascribed to the (102) and (110) crystal plane of monoclinic BiOBr (PDF# 09-0393), respectively. These results imply that the Cd^2+^ and Fe^3+^ do not change the main morphology of BiOBr. In addition, the element distribution corresponding with TEM (Figure 3c) was detected. As shown in Figure 3d–h, the Bi, O, Br, Cd, and Fe elements distributed well. The above results demonstrate that Cd^2+^ and Fe^3+^ were successfully synthesized in the BiOBr materials, respectively.

**Figure 4:**
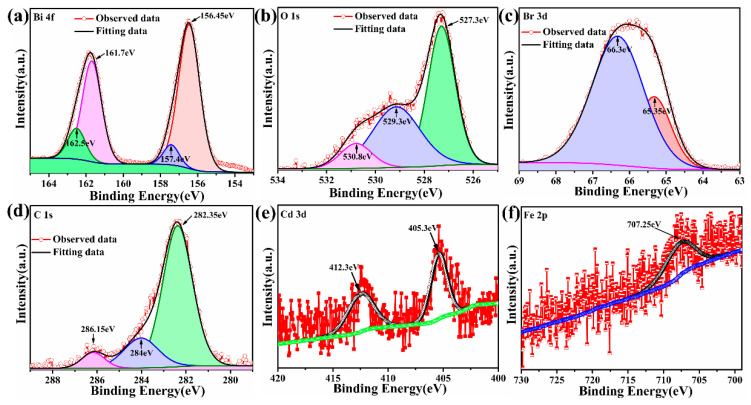
High-resolution XPS spectra of Fe/Cd co-doped BiOBr, Bi 4f spectrum (**a**), O1s spectrum (**b**), Br 3d spectrum (**c**), C 1s spectrum (**d**), Cd 3d spectrum (**e**) and Fe 2p spectrum (**f**).

The surface chemical composition of the BiOBr, Bi_1−*x*_Cd*_x_*OBr, Bi_1−*x*_Fe*_x_*OBr, Bi_1−*x*−*y*_Cd*_x_*Fe*_y_*OBr samples was investigated by XPS (Figure 4a–f. Figure S1 shows the survey of Fe/Cd co-doped BiOBr, indicating that Bi, O, Br, Fe, Cd, and C atoms, without other elements, existed on the surface of those samples. In the Bi 4f spectrum (Figure 4a), two peaks, locating at 156.45 and 161.7 eV, are assigned to the Bi 4f_7/2_ and Bi 4f_5/2_ of Bi^3+^ in Bi_1−*x*−*y*_Cd*_x_*Fe*_y_*OBr, respectively, which can be further fitted into two pairs of Bi^3+^ species coordinated with lattice oxygen (157.4 eV) and surface oxygen (162.5 eV) species [48]. The O 1 s spectrum in Bi_1−*x*−*y*_Cd*_x_*Fe*_y_*OBr (Figure 4b) is fitted into three peaks located at 527.3, 529.3, and 530.8 eV, associating with lattice oxygen, chemically adsorbed oxygen, and oxygen vacancy, respectively [49,50]. The peaks at 65.35 and 66.3 eV are attributed to Br 3d (Figure 4c) [48]. Three peaks located at 282.35, 284, and 286.15 eV are assigned to C1s (shown in Figure 4d); they are assigned to adventitious carbon species. The Cd 3d spectrum of Bi_1−*x*−*y*_Cd*_x_*Fe*_y_*OBr (Figure 4e) can be fitted into two peaks located at 405.3 and 412.3 eV, which can be regarded as contributions from Cd-O. The Fe 2p spectrum (Figure 4f) was seen at ~707.25 eV, which attributes Fe 2p^3/2^ to ferric (III) ions. Consequently, Fe and Cd atoms have successfully replaced Bi atoms into the lattice and formed stable structures with their stable valence states (Fe (III), Cd (II)).

**Figure 5:**
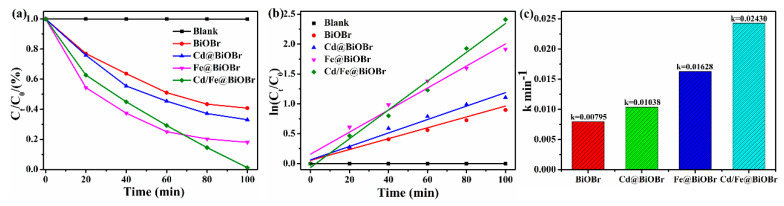
Variation of Rhodamine B (RhB) concentration (*C_t_*/*C*_0_) against photodegradation time for samples (**a**); pseudo-first-order kinetics for the degradation of RhB with BiOBr, Bi_1−*x*_Cd*_x_*OBr, Bi_1−*x*_Fe*_x_*OBr and Bi_1−*x*−*y*_Cd*_x_*Fe*_y_*OBr under visible light irradiation (**b**); ln(*C*_0_/*C_t_*) and irradiation time of samples (**c**).

The photocatalytic performance of products was evaluated by the photodegradation of Rhodamine B (RhB). Figure 5a shows the variation of RhB concentration (*C*/*C*_0_) with the photodegradation time of all samples. The photodegradation rates of RhB solution for BiOBr, Bi_1−*x*_Cd*_x_*OBr, Bi_1−*x*_Fe*_x_*OBr and Bi_1−*x*−*y*_Cd*_x_*Fe*_y_*OBr reached 59.3%, 66.9%, 81.9% and 98.8%, respectively. Obviously, the photocatalytic activity of Bi_1−*x*−*y*_Cd*_x_*Fe*_y_*OBr is the highest among all the specimens. The UV-vis spectra of the RhB solution are presented in Figure S2a–d. The results describe the gradient change of RhB concentration over pure-BiOBr and X-doped BiOBr (X = Cd, Fe, Cd/Fe) with irradiation time. The characteristic peak of as-prepared samples occurs at ~553 nm. Strangely, with the increase of irradiation time, the blueshift ~43.5 nm of the absorption peak of Bi_1−x_Cd_x_OBr can be found obviously, while the Bi_1−*x*_Fe*_x_*OBr does not. The reason for the relatively small blueshift (~20 nm) of Cd^2+^/Fe^3+^ co-doped BiOBr is that the blueshift is inhibited by Fe^3+^ ions, which illustrates the synergistic effect of Cd^2+^ and Fe^3+^ ions exists in the system. The peak intensity of doped samples decreased by increasing irradiation time, and the characteristic peak of Cd^2+^/Fe^3+^ co-doped BiOBr systems become almost disappeared after 100 min. It proves that the synergistic effect of Cd^2+^ and Fe^3+^ ions promote the degradation of organic pollutants.

The data of the photocatalytic performance test of the product accords with the pseudo-first-order model. The pseudo-first-order kinetic constant *k* is employed to evaluate the photodegradation rate (Figure 5b) to clarify the reaction kinetics of RhB [51]. The constants *k* of BiOBr, Bi_1−*x*_Cd*_x_*OBr, Bi_1−*x*_Fe*_x_*OBr and Bi_1−*x*−*y*_Cd*_x_*Fe*_y_*OBr were calculated to be 0.00795, 0.01038, 0.01628 and 0.02430 min^−1^, respectively (as shown in Figure 5c). The data demonstrate that the constants k for RhB by Bi_1−*x*−*y*_Cd*_x_*Fe*_y_*OBr are 3.10 times that of BiOBr. The kinetics study of the RhB dye has proved the ions matching and synergistic effect of Cd^2+^ and Fe^3+^ in the Bi_1−*x*−*y*_Cd*_x_*Fe*_y_*OBr photocatalysts; therefore, the photocatalytic performance on degrading RhB is improved greatly.

**Figure 6:**
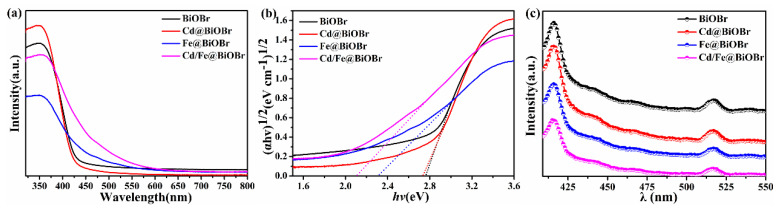
UV-vis spectra (**a**) and (*αhv*)^1/2^ vs. *hv* curves of photocatalysts (**b**); photoluminescence (PL) spectra (**c**).

The absorption spectra of products are shown in Figure 6a. The spectrum, responding at ~430 nm, indicating that BiOBr is excited by UV and visible light. The spectral absorption edge of Bi_1−*x*_Fe*_x_*OBr shows that a redshift of ~95 nm occurs, and the spectral absorption of Bi_1−*x*_Cd*_x_*OBr begins at 525 nm, which is earlier than that of BiOBr with the absorption begins at 475 nm. The redshift of ~145 nm could be observed obviously with the absorption edge of Bi_1−*x*−*y*_Cd*_x_*Fe*_y_*OBr. This result demonstrates that the absorption range was extended by Cd^2+^, Fe^3+^ ions doping in visible light. The Cd^2+^/Fe^3+^ co-doped BiOBr exhibits the strongest light absorption among samples. However, some drifts can be seen in the absorbance spectra (Figure 6a), which attributes to the specular reflection and hue of photocatalysts. Avoiding this spectral issue, the Kubelka–Munk equation, basing on Tauc plots of (*αhv*)^1/2^ versus energy (*hv*) (as shown in Figure 6b), was employed:

*αhv* = *A* (*hv* − Eg)^1/2^ (5)

In the formula, *α*, *h*, *v,* and *A* are absorption coefficient, Planck constant, optical frequency, and proportional constant, respectively [30,52,53,54]. In addition, the absorption edges and Eg of BiOBr, Bi_1−*x*_Cd*_x_*OBr, Bi_1−*x*_Fe*_x_*OBr, and Bi_1−*x*−*y*_Cd*_x_*Fe*_y_*OBr photocatalysts were calculated and listed in Table 1.

The photoluminescence (PL) spectra of pure-BiOBr and X-doped BiOBr (X = Cd, Fe, Cd/Fe) were obtained (Figure 6c). The PL data were employed to interpret the recombination rate of photogenerated e^−^ and h^+^, and the weaker PL intensity indicated the lower recombination efficiency [54,55,56,57,58,59]. Peaks at 416 and 517 nm were detected, as shown in Figure 6c. The PL intensity of doped BiOBr was dramatically lower than that of BiOBr, and that of Bi_1−*x*−*y*_Cd*_x_*Fe*_y_*OBr was the lowest among all samples. The results indicate that Cd^2+^ and Fe^3+^ ions repress the recombination of e^−^ and h^+^ and promote transferability carriers. In addition, according to the band structure results calculated by DFT, the impurity level was introduced into the BiOBr after doping of Fe^3+^ ions, which further contributed to the separation of e^−^ and h^+^. Consequently, the photocatalytic performance of BiOBr was promoted [60].

**Figure 7:**
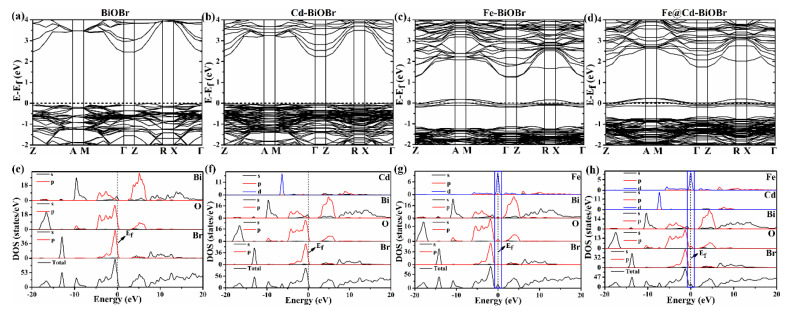
The density functional theory (DFT)-simulated band structure of BiOBr (**a**), Bi_1−*x*_Cd*_x_*OBr (**b**), Bi_1−*x*_Fe*_x_*OBr (**c**) and Bi_1−*x*−*y*_Cd*_x_*Fe*_y_*OBr (**d**); the density of states (DOS) of BiOBr (**e**), Bi_1−*x*_Cd*_x_*OBr (**f**), Bi_1−*x*_Fe*_x_*OBr (**g**) and Bi_1−*x*−*y*_Cd*_x_*Fe*_y_*OBr (**h**) crystals.

The electronic band structures of BiOBr, Bi_1−*x*_Cd*_x_*OBr, Bi_1−*x*_Fe*_x_*OBr, and Bi_1−*x*−*y*_Cd*_x_*Fe*_y_*OBr were obtained by DFT code to illustrate the Cd^2+,^ and Fe^3+^-doping effects (Figure 7). As shown in Figure 7a–d, the bandgap of pure-BiOBr, Bi_1−*x*_Cd*_x_*OBr, Bi_1−*x*_Fe*_x_*OBr and Bi_1−*x*−*y*_Cd*_x_*Fe*_y_*OBr was 2.45 eV, 2.24 eV, 2.39 eV and 2.38 eV, respectively. Noteworthily, the experimental values measured by the UV-vis spectrum (shown in Table 1) were larger than the theoretical ones. However, such deviation was common, especially when calculated values via the VASP (Vienna Ab-initio Simulation Package) code [61]. The bandgap of doped-BiOBr was smaller than that of BiOBr, which was in agreement with absorption spectra (Figure 6a). The electronic energy level of doped-BiOBr was abundant, which demonstrated additional electronic states occur near the Fermi level. This result could be attributed to some impurity energy levels [62,63]. The bandgap width of doped BiOBr occurred several notable changes: (1) the edge of the CB of Bi_1−*x*_Cd*_x_*OBr (as shown in Figure 7b) shifted ~0.22 eV towards the Fermi energy level; (2) the shift of ~1.2 eV occurred in the edge of the CB of Bi_1−*x*_Fe*_x_*OBr (Figure 7c) towards the Fermi energy level; (3) the VB of Bi_1−*x*_Fe*_x_*OBr shifted ∼1.14 eV away from the Fermi energy level; (4) the impurity energy levels from −0.205 to 0.17 eV (0.375 eV) emerged in the Bi_1−*x*_Fe*_x_*OBr. Comparing with the above results, the edge of the CB of Cd^2+^/Fe^3+^ co-doped BiOBr (Figure 7d) occurred the shift of ∼0.71 eV towards the Fermi energy level, and the VB of Bi_1−*x*_Fe*_x_*OBr shifted ~0.66 eV away from the Fermi energy level. The impurity energy levels were broadened from 0.375 (Fe-doping) to 0.42 eV (from 0.186 to 0.234 eV). Consequently, in the Cd^2+^/Fe^3+^ co-doped BiOBr system, the contribution of Cd^2+^ ions for electrons was mainly located at the top of the valence band, and the contribution of Fe^3+^ ions for electrons was providing the impurity energy band of ~0.42 eV (from 0.186 to 0.234 eV). With the synergistic effect of Cd^2+^ and Fe^3+^ ions, the bandgap of the sample was reduced, and the energy level of the impurities was provided, thereby achieving effective separation of electrons and holes. The excellent photocatalytic performance of as-prepared products, owning a narrow bandgap, and impurity energy levels could be obtained easily [64]. Moreover, Bi_1−*x*−*y*_Cd*_x_*Fe*_y_*OBr were indirect bandgap semiconductors, which is confirmed in Figure 7. The indirect semiconductor properties are beneficial to improve the photocatalytic activity [65].

The total and part of the density of states (TDOS and PDOS) of BiOBr, Bi_1−*x*_Cd*_x_*OBr, Bi_1−*x*_Fe*_x_*OBr, and Bi_1−*x*−*y*_Cd*_x_*Fe*_y_*OBr are shown in Figure 7e–h, which can be applied investigating the effects of the Cd^2+^ and Fe^3+^ ions of BiOBr photocatalyst. The Br *4p*, *6p,* and O *2p* orbitals in BiOBr (as shown in Figure 7e) were major components at −8–0 eV in the VB region. The O *2p* and Br *4p* orbitals play the main role in contributing electrons. The Bi *6s* and *6p* contribute the most electrons in the CB region. The electronic structures, including the impurity state and the localized doped band existing near the Fermi energy level, are shown in Figure 7f–h.

The CB of Bi_1−*x*_Cd*_x_*OBr mainly consists of Cd *3s* + Cd *3p* + Bi *6p* + O *2p* orbital (Figure 7f). The Cd *3d* + Bi *6p* + O *2p* hybrid orbitals are the main part of states in the VB region from −8 to 0 eV, and the states of the electronic contribution of Cd *3d* hybrid orbital is ~−6 eV. Corresponding to the result of the energy band structure, the energy level at −6 eV of the VB is denser and flatter than that of pure-BiOBr. The CB of Bi_1−*x*_Fe*_x_*OBr is made up with Fe *3p* + Fe *3d* + Bi *6p* + O *2p* hybrid orbital (Figure 7g), and the Fe *3d* hybrid orbital contributes more electrons. After doping Fe^3+^, the shift of the Fermi energy level can be observed towards the minimum conduction-band. Additionally, the charges transfer from Fe *3d* to Fe *3s* + Fe *3d* + Bi *6p* + O *2p* orbitals (As shown a dotted box in Figure 7g). Therefore, defect states at 0 eV generate within the bandgap of BiOBr. Compared with pure BiOBr, the electronic structure of Bi_1−x_Fe_x_OBr has an impurity state of 0 eV, and there is a local Fe3d energy band in its energy band structure. Moreover, when Cd^2+^ and Fe^3+^ are doping into the BiOBr (Figure 7h), the CB of Bi_1−*x*−*y*_Cd*_x_*Fe*_y_*OBr is made up with Fe *3p* + Fe *3d* + Bi *6p* + O *2p* orbitals. The Fermi energy level occurs the shift towards the minimum valence-band, which attributes to the doping of Cd^2+^ ions. The fact that charges transfer from Fe *3d* to Fe *3s* + Fe *3d* + Bi *6p* + O *2p* orbitals (As shown in Figure 7h with the dotted box) is promoted by the contribution electrons of Cd^2+^ ions to valence band electrons in the Bi_1−*x*−*y*_Cd*_x_*Fe*_y_*OBr. Consequently, with the synergistic effect of Cd^2+^ and Fe^3+^ ions, the photogenerated e^−^/h^+^ pairs can be separated effectively. The separated electrons and holes play different roles at CB and VB, completing the photodegradation.

**Figure 8:**
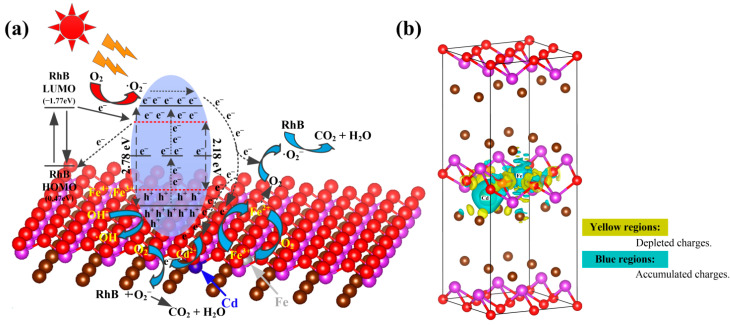
Proposed mechanism of photocatalytic activity by Bi_1−*x*−*y*_Cd*_x_*Fe*_y_*OBr photocatalyst (**a**); calculated charge density difference of Bi_1−*x*−*y*_Cd*_x_*Fe*_y_*OBr (**b**).

The possible degradation mechanism of Bi_1−*x*−*y*_Cd*_x_*Fe*_y_*OBr as photocatalyst was investigated (Figure 8). The *E*_VB_ of BiOBr surpassed the reaction potential of OH^−^/•OH (2.38 eV) [66], indicating that the •OH radicals could form by reacting h^+^ with OH^−^ ions. The LUMO (Lowest Unoccupied Molecular Orbital) of RhB (−1.77 eV) [67] was negative: electrons from RhB migrate into the CBMs of products, and some of them react with O_2_, and •O_2_^−^ radicals could be obtained. Moreover, the HOMO (Highest Occupied Molecular Orbital) of RhB (~0.47 eV) Inhibition of •O_2_^−^ formation (−0.05 eV), implying that •O_2_^−^ reacts by h^+^, which comes from HOMO, the e^−^ were again migrated into HOMO (as shown in Figure 8a).

As for RhB, it is well-known that the degradation process of RhB includes: N-demethylation and destruction of conjugated structure [68]. The results of the experiment were analyzed synthetically, and the peak at ~553 nm is interpreted as effects from the chromosphere structure of RhB. The absorption peak decreased by increasing irradiation time, indicating that the process of destruction of the conjugated structure occurred in the system. Moreover, the absorption peak of pure BiOBr and Fe^3+^-doped BiOBr show no obvious blueshift, demonstrating that the N-demethylation process of RhB did not occur [69]. However, the blueshift of ~42 nm and 23 nm occurred in the absorption peak of Cd^2+^-doped BiOBr and Cd^2+^/Fe^3+^ co-doped BiOBr, respectively, implying that the N-demethylation process of RhB happened by Cd^2+^ ions during the reaction. Consequently, it can be concluded that the synergistic effect of Cd^2+^ and Fe^3+^ ions would have a positive influence on the degradation processes of RhB in the BiOBr photocatalyst.

From the perspective of photocatalysts, the morphology of Bi_1−*x*−*y*_Cd*_x_*Fe*_y_*OBr (shown in Figure 1d) showed that the flowerlike nanospheres are self-assembled from ultra-thin nanoflakes, which is consistent with Scheme 1. A large number of active sites can be found in the products, which can increase the probability of catalytic reactions. In addition, the electrons in Bi_1−*x*−*y*_Cd*_x_*Fe*_y_*OBr can be excited easier than BiOBr. The Eg of Cd doped BiOBr is smaller than that of BiOBr, and the Fermi level of Cd is lower than that of BiOBr; therefore, the photogenerated electrons occurred movement toward Cd, attributing to the formation of the Schottky barrier [70,71]. Second, the photogenerated electrons are transferred to the surface of Bi_1−*x*−*y*_Cd*_x_*Fe*_y_*OBr to participate in the reduction reactions. Third, The Fe atom replaced the Bi atom into the BiOBr lattice and existed as metastable Fe^3+^ ions. Meanwhile, Fe^3+^ ions can further form Fe^2+^ and Fe^4+^ ions by capturing e^−^ and h^+^ during photocatalysis. However, the Fe^2+^ and Fe^4+^ ions with six and four electrons in the 3d orbital are not stable in the catalytic system. Therefore, the charges captured by Fe^2+^ and Fe^4+^ will easily release and migrate to the surface of Bi_1−*x*−*y*_Cd*_x_*Fe*_y_*OBr to participate in the catalytic reaction. The Fe^2+^ ions are oxidized and converted to the metastable Fe^3+^ ions on the surface of Bi_1−*x*−*y*_Cd*_x_*Fe*_y_*OBr. The •O_2_^−^ radicals can be converted from O_2_ by obtaining electrons. Meanwhile, Fe^4+^ ions are converted to metastable Fe^3+^ ions [72]. The trapped holes play an indispensable role in decomposing RhB dye in the catalytic system. Thereby the electrons and the holes can be separated, and more •OH radicals form onto the Bi_1−*x*−*y*_Cd*_x_*Fe*_y_*OBr surface due to the higher concentration of holes. Because of photogenerated electrons, more •O_2_^−^ free radicals can be formed. The emerging •OH and •O_2_^−^ radicals would foster reactions. The •OH radicals with positive electronic states prefer capturing the holes from the VB. The electrons in the CB can induce the formation of photogenerated •OH radicals.

Consequently, the synergistic effect of the Cd^2+^ and Fe^3+^ ions reduced the bandgap width of BiOBr photocatalyst and provided impurity energy levels for the electronic transition. As a result, the electrons and holes can be separated efficiently and participated in the photocatalytic reaction. The detailed reaction processes are the following:

BiOBr/RhB + *hv* → BiOBr/RhB (e^−^ + h^+^) (6)

Fe^3+^ + e^−^ → Fe^2+^ (7)

Fe^2+^ + O_2_ → Fe^3+^ + •O_2_^−^ (8)

O_2_ + Fe^3+^ + e^−^ → Fe^2+^ (9)

Fe^3+^ + h^+^ → Fe^4+^ (10)

Fe^4+^ + e^−^ → Fe^3+^ (11)

OH^−^ + h^+^_VB_ → •OH (12)

O_2_ + e^−^_CB_ → •O_2_^−^ (13)

h^+^ or •O_2_^−^ + RhB → CO_2_ + H_2_O (14)

Furthermore, the calculated charge density difference of Bi_1−*x*−*y*_Cd*_x_*Fe*_y_*OBr photocatalysts was calculated to investigate the distribution of charges on the surface of Cd and Fe atoms. As shown in Figure 8b, many blue regions can be seen around the Fe and Cd atoms; more precisely, the Fe and Cd atoms are completely surrounded. These regions represent the accumulated charges in the bonding electron coupling process. The accumulated charges of the Cd atom are concentrated relatively, while that of the Fe atom is dispersed. The result indicates that the outermost electrons of the Fe atom are easy to exchange and move with the outermost electrons of O atoms surround it. In addition, the yellow regions in Figure 8b represent the depleted charges in the bonding electron coupling, which are mainly located around the O and Br atoms adjacent to the Cd and Fe atoms. Consequently, the charge transfer between doped atoms and their surrounding atoms (O and Br atoms) is realized in different ways, especially the exchange between Fe atoms and the outermost electrons of the surrounding O atoms, forming a high-speed moving public electron band, which is expected to promote the effective separation and transfer of e^−^/h^+^ species in the catalytic system. Additionally, the calculated charge density difference of Bi_1−*x*_Cd*_x_*OBr and Bi_1−*x*_Fe*_x_*OBr is provided in supporting materials (Figure S3). In conclusion, the synergistic effect of Cd^2+^ and Fe^3+^ plays a key role in promoting the transfer of electrons on the surface of materials.

**Figure 9:**
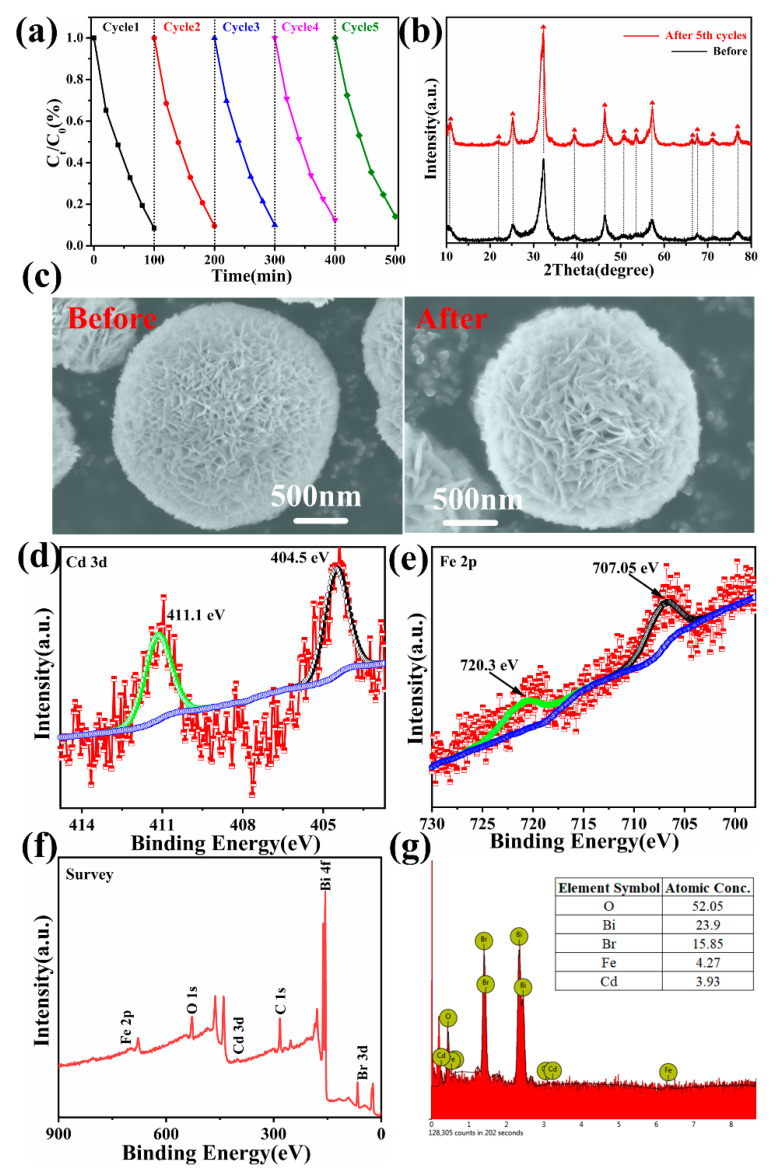
Recycle experiments of Bi_1−*x*−*y*_Cd*_x_*Fe*_y_*OBr photocatalysts for photodegraded RhB (**a**); XRD patterns (**b**); SEM images (**c**); high-resolution XPS spectra: Cd 3d spectrum (**d**), Fe 2p spectrum (**e**) and survey spectrum (**f**); EDS spectrum (**g**) of after the photocatalytic cyclic reactions.

The Bi_1−*x*−*y*_Cd*_x_*Fe*_y_*OBr photocatalyst was cycled five times under the same reaction conditions to investigate the photocatalytic stability. The result of relatively stable photocatalytic performance is shown in Figure 9a. It can be seen that Bi_1−*x*−*y*_Cd*_x_*Fe*_y_*OBr photocatalyst still retains a relatively high photocatalytic performance, with catalytic efficiency of 86%, for the RhB degradation after five cycles. The sedimentation and the transferring processes can be a major reason for the decreased photocatalytic performance. Figure 9b shows XRD patterns of Bi_1−*x*-*y*_Cd*_x_*Fe*_y_*OBr photocatalyst before and after the photocatalytic cyclic reactions, indicating that the phase has no significant change after the photocatalytic cyclic reaction. Figure 9c shows SEM images of Bi_1−*x*−*y*_Cd*_x_*Fe*_y_*OBr photocatalyst before and after the photocatalytic cyclic reactions, which indicates the microstructure has no significant change after the photocatalytic cyclic reaction. Figure 9d–f shows High-resolution XPS spectra of Cd^2+^ and Fe^3+^ and survey spectrum of Bi_1−*x*−*y*_Cd*_x_*Fe*_y_*OBr photocatalysts after the photocatalytic cyclic reactions. The EDS spectrum of Bi_1−*x*−*y*_Cd*_x_*Fe*_y_*OBr photocatalysts after cycle test shown in Figure 9g indicates that the chemical composition of the photocatalysts has no changes. Therefore, the Bi_1−*x*−*y*_Cd*_x_*Fe*_y_*OBr photocatalyst shows a highly efficient photocatalytic activity and outstanding recyclability for the degradation of RhB dye under visible light irradiation.

## 4. Conclusions

The uniform flowerlike microspheres Cd^2+^/Fe^3+^ co-doped BiOBr photocatalysts, assembled by ultrathin nanosheets, were synthesized by hydrothermal synthesis. The results demonstrate that the Cd^2+^-doping and Fe^3+^-doping can promote the photocatalytic performance of BiOBr to a certain extent by reducing the bandgap and introducing the impurity band, respectively. Moreover, the novel Cd^2+^/Fe^3+^ co-doped BiOBr photocatalysts presented a remarkably photocatalytic performance for the decomposition of organic dyes, comparing with pure BiOBr, by achieving synergistic effects of the enhanced photogenerated e^−^/h^+^ separation and narrowed the bandgap with the ions synergistic effect of Cd^2+^ and Fe^3+^. The reasonable growth mechanism and catalytic mechanism confirmed the rationality of the experimental results, which shows that the synergistic effects of multi-ion doping have great potential in the field of photocatalysis.

## Figures and Tables

**Table 1:** Absorption edges and band gaps for photocatalysts.

Sample	Absorption Edge (nm)	Bandgap (eV)	
		Theoretical Values	Experimental Values
BiOBr	427.5	2.45	2.78
Cd@BiOBr	431.5	2.24	2.74
Fe@BiOBr	462.5	2.39	2.31
Cd/Fe@BiOBr	484	2.38	2.12

## Data Availability

All data are presented in the form of charts in the article.

## Supplementary Materials

The following are available online at https://www.mdpi.com/2079-4991/11/2/423/s1, Figure S1. The survey spectra of Bi1-x-yCdxFeyOBr. Figure S2. The UV–vis spectral of RhB solution of BiOBr (a), Bi1-xCdxOBr (b), Bi1-xFexOBr (c) and Bi1-x-yCdxFeyOBr (d). Figure S3. The calculated charge density difference of Bi1-xFexOBr (a) and Bi1-xCdxOBr (b).
