# Supplementary material for: New Insights into Cd2+/Fe3+ Co-Doped BiOBr for Enhancing the Photocatalysis Efficiency of Dye Decomposition under Visible-Light
Source: Nanomaterials (Basel). 2021 Feb 7;11(2):423. doi: 10.3390/nano11020423 (PMC7914912; doi:10.3390/nano11020423)
Supplement: Supplementary file 1 [file nanomaterials-11-00423-s001.pdf]

Supporting Information

Article

New Insights into Cd^{2+} /Fe^{3+} Co-Doped BiOBr for Enhancing the Photocatalysis Efficiency of Dye Decomposition under Visible-Light

Hong Sheng [1,†], Wei Wang [2,3,†], Rong Dai [2], Jing Ning [2], Lei Zhang [2], Qiao Wu [2], Fuchun Zhang [2,*], Junfeng Yan [3,*] and Weibin Zhang [4,*]

[1] College of Mathematics & Physics, Weinan Normal University, Weinan 714000, China; wnshenghong@163.com
[2] School of Physics and Electronic Information, Yan'an University, Yan'an 716000, China; wangwei@yau.edu.cn (W.W.); dairong@yau.edu.cn (R.D.); ningjing@yau.edu.cn (J.N.); yadxzl960203@163.com (L.Z.); wq@yau.edu.cn (Q.W.)
[3] School of Information Science Technology, Northwest University, Xi'an 710127, China
[4] School of Physics and Optoelectronic Engineering, Yangtze University, Jingzhou 434023, China
* Correspondence: yadxzfc@yau.edu.cn (F.Z.); yanjf@nwu.edu.cn (J.Y.); zhang@yangtzeu.edu.cn (W.Z.). Tel.: +86-1832-991-8036 (F.Z.); +86-1362-928-0982 (J.Y.); +86-1311-719-0486 (W.Z.)
† Hong Sheng and Wei Wang contributed equally.

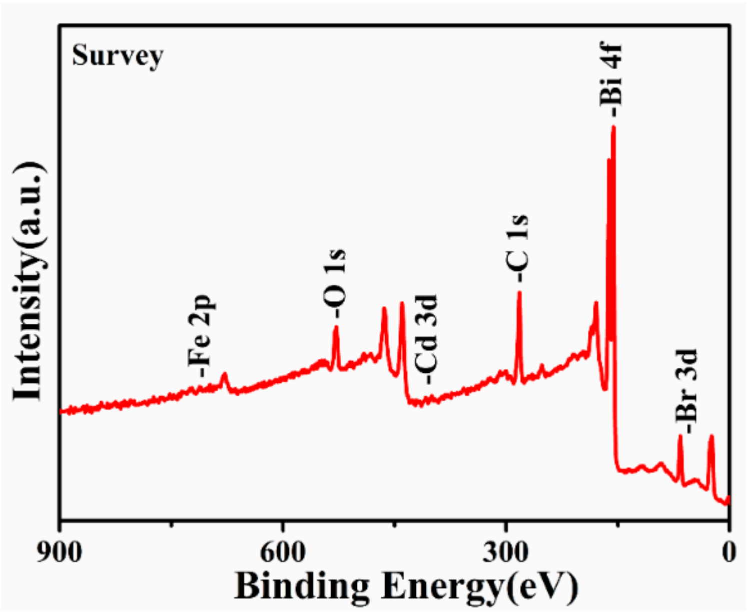

Figure S1. The survey spectra of $Bi_{1-x-y}Cd_xFe_yOBr$.

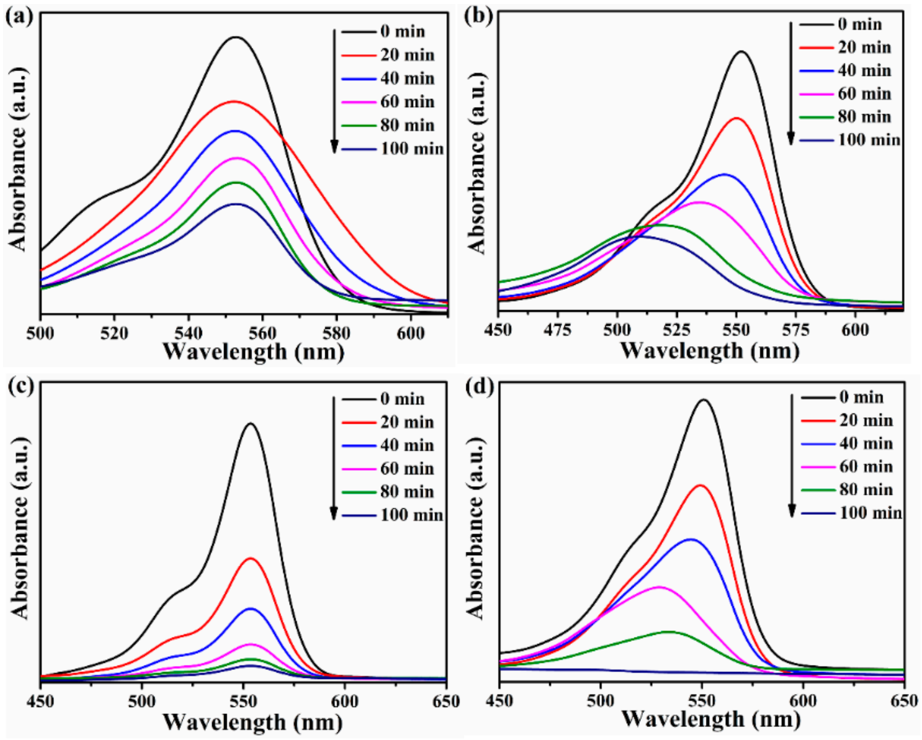

Figure S2. The UV–vis spectral of RhB solution of BiOBr (**a**), $Bi_{1-x}Cd_xOBr$ (**b**), $Bi_{1-x}Fe_xOBr$ (**c**) and $Bi_{1-x-y}Cd_xFe_yOBr$ (**d**).

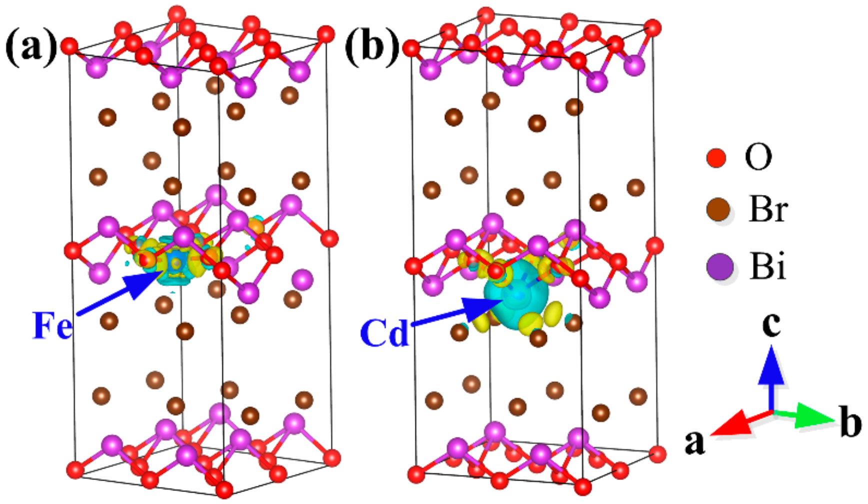

Figure S3. The calculated charge density difference of $Bi_{1-x}Fe_xOBr$ (a) and $Bi_{1-x}Cd_xOBr$ (b).
